# Chondroitin Sulfate Promotes the Proliferation of Keloid Fibroblasts Through Activation of the Integrin and Protein Kinase B Pathways

**DOI:** 10.3390/ijms21061955

**Published:** 2020-03-13

**Authors:** Yasuhiro Katayama, Motoko Naitoh, Hiroshi Kubota, Satoko Yamawaki, Rino Aya, Toshihiro Ishiko, Naoki Morimoto

**Affiliations:** 1 Department of Plastic Surgery, Graduate School of Medicine, Kyoto University, 54 Shogoin-Kawaharacho, Sakyo-ku, Kyoto City, Kyoto 606-8507, Japan; hemim@kuhp.kyoto-u.ac.jp (Y.K.); mnaoki22@kuhp.kyoto-u.ac.jp (N.M.); 2 Department of Plastic Surgery, Kobe City Medical Center General Hospital, 2-2-1, Minatojimaminamimachi, Chuo-ku, Kobe City, Hyogo 650-0047, Japan; 3 Riken Center for Developmental Biology, 2-2-3 Minatojimaminamimachi, Chuo-ku, Kobe City, Hyogo 650-0047, Japan; 4 Department of Life Science, Faculty of Engineering Science, Akita University, 1-1 Tegata Gakuenmachi, Akita 010-8502, Japan; hkubota@gipc.akita-u.ac.jp; 5 Department of Plastic and Reconstructive Surgery, Japanese Red Cross Fukui Hospital, 2-4-1, Tsukimi, Fukui City, Fukui 918-8501, Japan; satokoy@kuhp.kyoto-u.ac.jp; 6 Department of Plastic Surgery, Kyoto Katsura Hospital, 17-banchi, Yamada Hirao-cho, Nishikyo-ku, Kyoto-shi, Kyoto 615-8256, Japan; rinok@kuhp.kyoto-u.ac.jp; 7 Department of Plastic Surgery, Japanese Red Cross Otsu Hospital, 1-1-35, Nagara, Otsu City, Shiga 520-0000, Japan; ishiko@otsu.jrc.or.jp

**Keywords:** fibroblast, keloids, chondroitin sulfate, PI3K/Akt pathway, integrin

## Abstract

Keloids are dermal fibroproliferative tumors that arise beyond the boundary of the original wound edges and invades adjacent tissue. Keloids are characterized by the extensive production of extracellular matrix (ECM) and abnormal fibroblast proliferation. Chondroitin sulfate (CS) is one of the major structural components of cartilage and ECM. Recently, we reported the over-accumulation of CS in keloid lesions. Keloid-derived fibroblasts (KFs) and normal dermal fibroblasts (NFs) were incubated with CS. The fibroblast proliferation rate was analyzed using a tetrazolium salt colorimetric assay. The activation of the intracellular signaling pathway was analyzed by Western blotting. Wortmannin, a PI3K inhibitor, and anti-integrin antibodies were tested to investigate the mechanism of the CS-induced cell proliferation. CS strongly stimulated the proliferation of KFs, but not NFs. The analysis of the intracellular signal transduction pathway revealed that the stimulation effect of CS on KF proliferation was due to the activation of the protein kinase B (AKT) pathway and that integrin α1 was responsible for this phenomenon. We revealed that CS probably activates the AKT pathway through integrin to induce KF proliferation. CS may be a novel clinical therapeutic target in keloids.

## 1. Introduction

Keloids are benign dermal fibroproliferative tumors that grow beyond the boundary of the original wound edge and invade the adjacent tissue due to the extensive production of extracellular matrix and the abnormal proliferation of fibroblasts [1]. Keloids arise spontaneously or during the wound healing process of the dermis, and rarely regress without treatment. The treatment of keloids has long been a major problem for plastic surgeons due to the high rate of recurrence.

Multimodal treatments, including surgical excision, radiotherapy, corticosteroid injection, physical therapy, and combinations of these treatments, have been applied. Still, there is no satisfactory treatment. The mechanism underlying keloid formation remains unclear. Thus, revealing the etiology of keloids is a fundamental step in the development of clinical treatments for the condition.

Recently, the formation of keloids has been recognized to occur due to the excess proliferation of fibroblasts with the over-accumulation of the extracellular matrix. Fibroblasts cultured from keloids (KFs) show altered behavior from normal dermal fibroblasts (NFs), including rapid proliferation, excessive extracellular matrix production, and increased migration activity, which may be a significant etiological factor in keloid development.

To understand the behavior of KFs, we studied molecular signal transduction pathways, including growth factor-induced cell growth, apoptosis, and intracellular signal transduction [2,3,4,5]. KFs have been reported to show the overexpression of insulin-like growth factor 1 [6,7,8,9] and altered epigenetic patterns [10], which have been considered as causes of the invasive and proliferative behavior of KFs.

Chondroitin sulfate (CS) is a glycosaminoglycan (GAG) composed of alternating, differently-sulfated residues of β-D-glucuronate and β-D-N-acetylgalactosamine [11]. CS is one of the structural molecules of the extracellular matrix, which is present in high levels in the cartilage and dermis. CS chains are classified into types A, B, C, D, E, K, and H [12], according to the specific sulfation pattern. We focused on CS not only as a structural molecular scaffold but also as the functional component of the extracellular milieu. In human fibroblasts, CS A and CS C promote wound closure in vitro and stimulate cellular adhesion and proliferation [11]. Human wound fluid contains glycosaminoglycans in high concentrations [13]. These studies suggest that CS may play an essential role beyond its role in the formation of the ECM structure.

Our previous study showed that Chondroitin sulfate was over-accumulated in keloids [14] and that the degradation of CS was associated with pathological improvement in keloids [15]. However, the mechanism through which chondroitin sulfate is involved in the etiology of keloids has not been investigated well. To investigate how CS is involved in the enhanced KF proliferation, we studied intracellular signaling events by Western blotting of signaling molecules.

## 2. Results

### 2.1. Chondroitin Sulfate Stimulates the Proliferation of Keloid-Derived Fibroblasts

We explored the novel roles of CS in the positive regulation of KF proliferation by focusing on the promotion of the CS-mediated signaling pathway. This was directed by our previous study, which suggested that the over-accumulation of CS in keloid lesions may be the deteriorating etiological factor [14,15]. Recently, the involvement of CS in wound healing has been reported [11,16,17,18]. We hypothesized that the over-accumulated CS might be a vital molecule in abnormal wound healing and keloids. In an attempt to elucidate how CS affects KF behavior in vitro, we compared KFs and NFs in a cellular proliferation assay and analyzed the intracellular signaling pathways.

**Figure 1:**
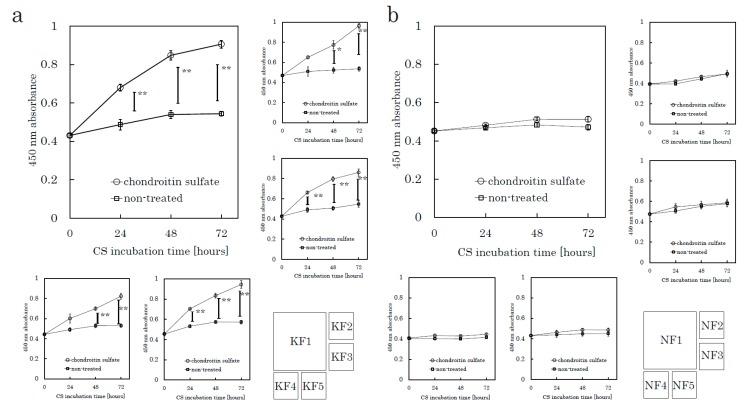
CS stimulates keloid fibroblast (KF) proliferation but not normal fibroblast (NF) proliferation. KFs and NFs were cultured with chondroitin sulfate (CS) or without CS. Cell proliferation was analyzed using a colorimetric assay with a water-soluble tetrazolium salt as the substrate. Proliferation curves of KFs (K1 to K5, Table 1) (**a**) and NFs (N1 to N5, Table 1) (**b**) are shown. Error bars represent the standard deviation (*n* = 3). * *p* < 0.05, ** *p* < 0.01.

The following assays were conducted to evaluate CS-mediated cell proliferation. We used culture plates precoated with poly-D-Lysine (PDL), which is used as a substrate to support the maintenance of negatively charged CSs. Fibroblasts were harvested from tissue specimens using the explant method. We seeded fibroblasts onto PDL plates and incubated the plates for 24 h in serum-free media before CS stimulation. We analyzed five KFs and five NFs in total. A colorimetric tetrazolium salt assay revealed that CS significantly stimulated KF proliferation in comparison to the control (no CS) group (Figure 1 and Supplementary Figure S1a). CS did not stimulate the proliferation of NFs. Even though we did not add fetal bovine serum (FBS) in this experiment, CS-stimulated KFs showed significant cellular proliferation. These distinct characteristics suggested that the mechanisms underlying the CS-promoted proliferation in KFs might differ from those in NFs. 

### 2.2. CS Promotes the Proliferation of KFs by Activating the Protein Kinase B (AKT) Pathway

**Figure 2:**
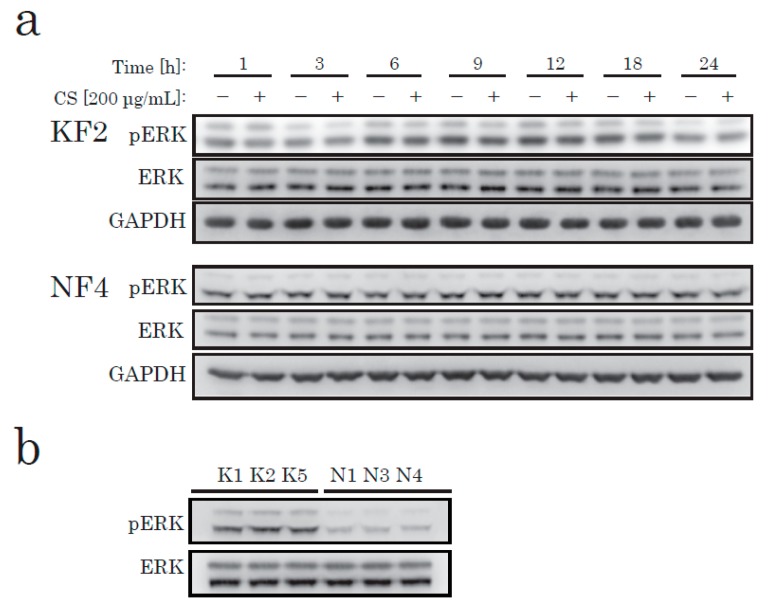
Western blotting of phospho-ERK (pERK) and total ERK (ERK) in KFs and NFs. KFs and NFs were treated with CS for up to 24 h. Soluble protein extract (8 µg/lane) was analyzed using antibodies specific to pERK, ERK, or glyceraldehyde-3-phosphate dehydrogenase (GAPDH). (**a**) KFs from K2 and NFs from N4 (Table 1) were analyzed. (**b**) KFs and NFs from three different patients were analyzed.

To elucidate the effects of CS on KFs in cell proliferation, we next identified the canonical pathways that have been shown to alter the activation of KFs from recent studies [19,20], such as the MAPK/ERK, JNK, and AKT/PI3K pathways. To determine the pathway that produces the signaling to induce CS-mediated proliferation in KFs, we investigated the change in the intracellular signaling pathways after CS stimulation. According to our data, CS stimulation caused no significant change in phosphorylated ERK in either KFs or NFs (Figure 2a). However, in comparison to NFs, ERK was highly phosphorylated in KFs under starved conditions (Figure 2b).

**Figure 3:**
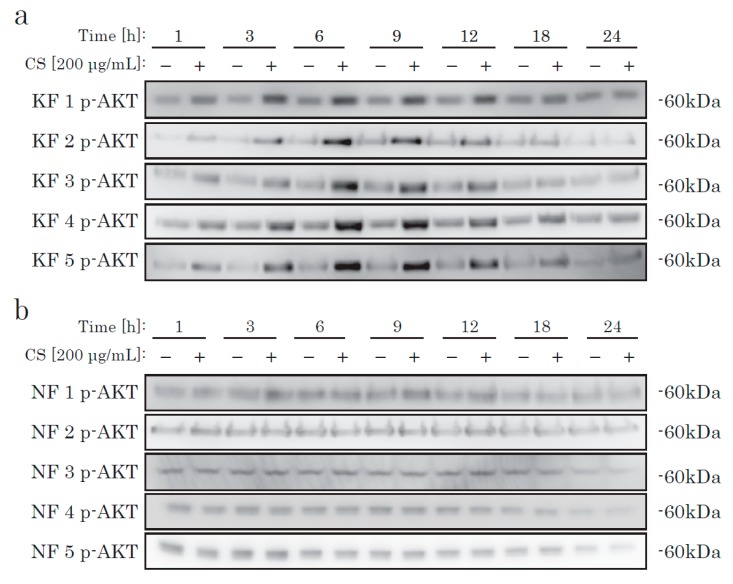
The time-dependent phosphorylation of AKT in KFs cultured with CS. KFs (**a**) and NFs (**b**) were incubated with CS and analyzed by Western blotting using an antibody specific to phosphorylated AKT.

We then checked AKT activation. In KFs, incubation with CS elevated the phosphorylation level of AKT, in a time-dependent manner (Figure 3). In NFs, we could not find any difference in the AKT activation level between the CS and control groups. Thus, we hypothesized that there is a CS-specific receptor-like mechanism that occurs via the AKT pathway in KFs, but not in NFs. 

**Figure 4:**
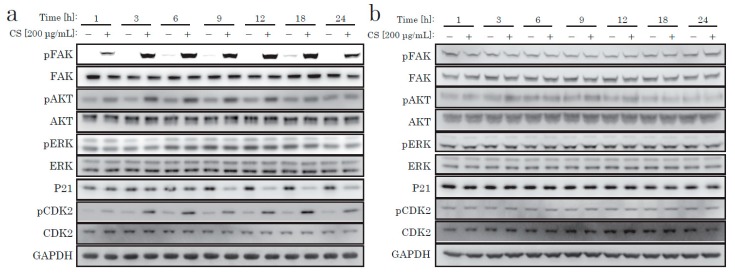
Western blotting of proteins regulating the intracellular signaling pathway. KFs (**a**) and NFs (**b**) were treated with CS for up to 24 h.

Next, we focused on the AKT pathway change caused by the CS stimulation. We examined downstream signal proteins related to the cell cycle. The cell cycle progression from G0 to S phase requires several cell-cycle regulating molecules in specific states, such as decreased p21, activated CDK2, and increased cyclin D. p21 is a cyclin-dependent kinase inhibitor (CKI) that decreases when the cell cycle progresses. A few hours after CS stimulation, we discovered the time-dependent phosphorylation of AKT, decreased p21, and CDK2 phosphorylation (Figure 4). In contrast, NFs did not display such responses after CS stimulation (Figure 4 and Supplementary Figure S1b). We concluded that the cell proliferative effect of CS on KF was due to the activation of the AKT pathway. 

**Figure 5:**
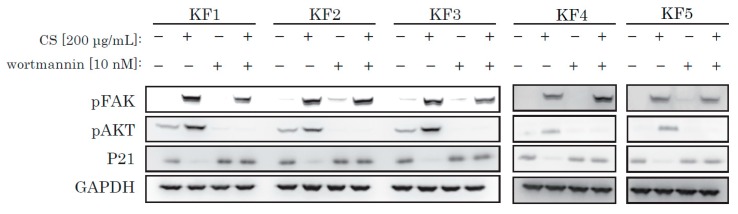
Wortmannin blocked the CS-induced activation of AKT and also blocked a decrease of p21. KFs were incubated with CS and wortmannin and analyzed by Western blotting.

To reinforce our theory, we used wortmannin, a selective and irreversible PI3K/Akt inhibitor. We incubated KFs with wortmannin and obtained protein samples. Wortmannin successfully blocked CS-mediated activation of AKT and blocked p21 decrease (Figure 5), and inhibited CS-induced activation of KF proliferation (Supplementary Figure S2). 

### 2.3. CS Promotes the Proliferation of KFs via a Specific Type of Integrin

From recent research [21] and our own data, we hypothesized that integrin might act as a receptor for CS stimulation. The major downstream signaling molecule after integrin is FAK (focal adhesion kinase). FAK is a non-receptor tyrosine kinase, which is activated on the binding of integrin to ECM and which generates downstream signal transduction. As shown in Figure 4 and Figure 5, we only observed robust phosphorylation of FAK when CS was blocked by wortmannin, which prevented the decrease of p21. Throughout the experiment, CS activated FAK. Thus, the stimulation effect of CS on KF proliferation was possibly due to the activation of the FAK and the AKT pathways.

**Figure 6:**
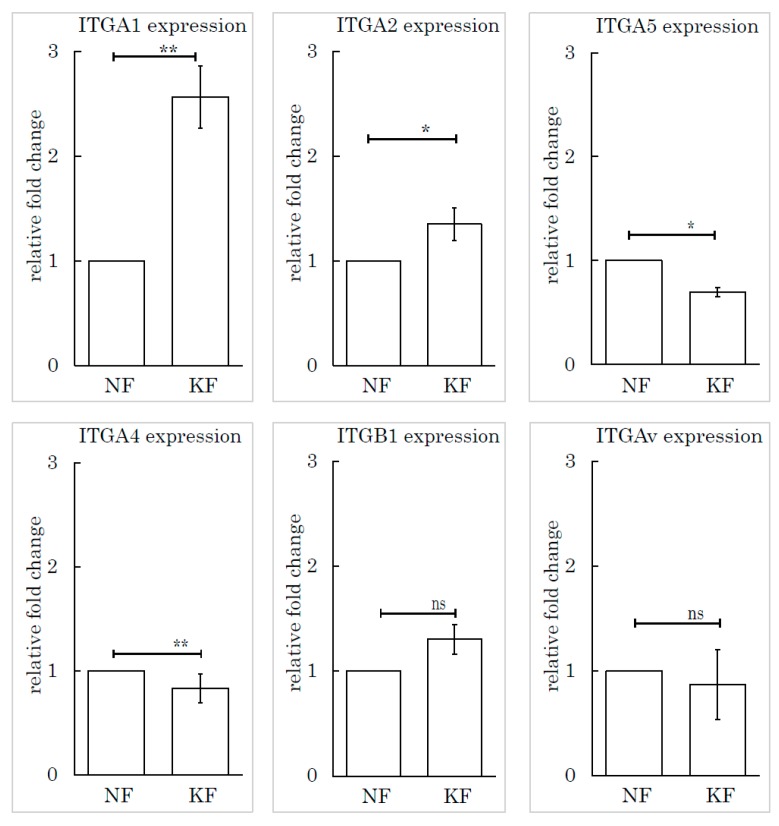
The integrin α1 expression level in KFs was high relative to NFs. Real-time PCR was performed to analyze the integrins (ITGA1, ITGA2, ITGA4, ITGA5, ITGB1, and ITGAv) in KFs and NFs. ** *p* < 0.01, * *p* < 0.05, ns indicates not significant, *n* = 5 (KFs), 7(NFs).

To determine which subtype of integrin was responsible for this mechanism, we selected and investigated several subtypes of integrin based on the relevant literature: integrin α1, integrin α2, integrin α4, integrin α5, integrin β1, and integrin Av. We measured the expression levels of these subtypes by RT-PCR. Integrin α1 (ITGA1) was highly expressed in KFs, relative to NFs (Figure 6). 

**Figure 7:**
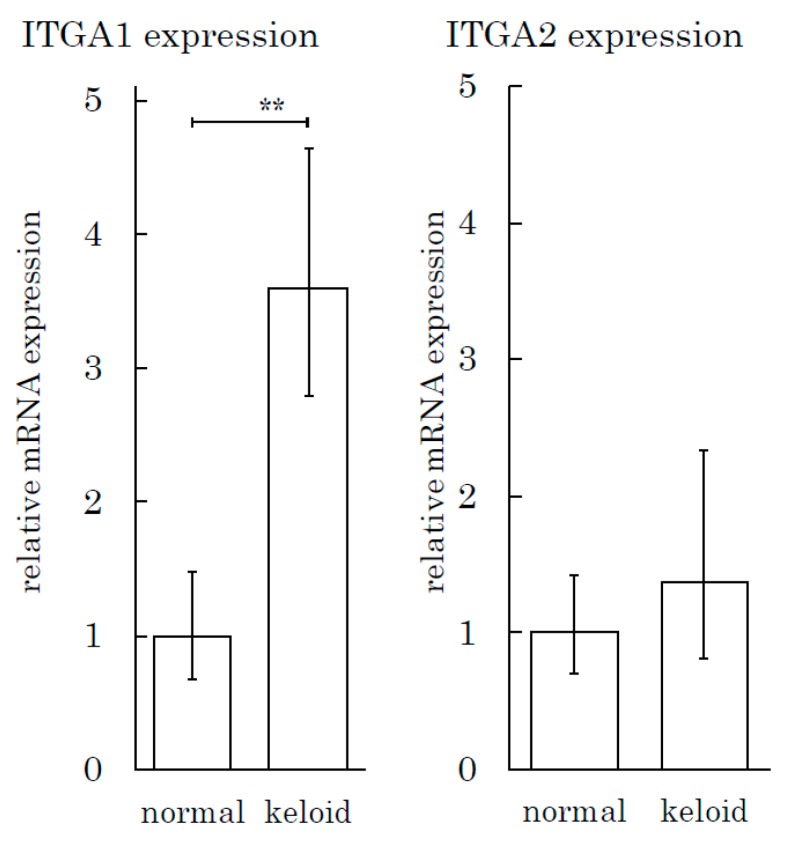
The Integrin α1 expression level is high in keloid tissue relative to normal skin tissue. The real-time PCR analysis of integrins (ITGA1 and ITGA2) was performed for keloid and normal skin tissue samples. ** *p* < 0.01. *n* = 5 (KFs), 7(NFs).

We also conducted an integrin expression assay to measure the integrin expression in tissue specimens. The expression of ITGA1 in keloids was three times greater than that in normal skin tissue (Figure 7). The ITGA2 level in keloids and normal skin tissues did not differ to a statistically significant extent. 

### 2.4. Anti-Integrin α1 Antibody Blocks CS-Induced KF Proliferation 

**Figure 8:**
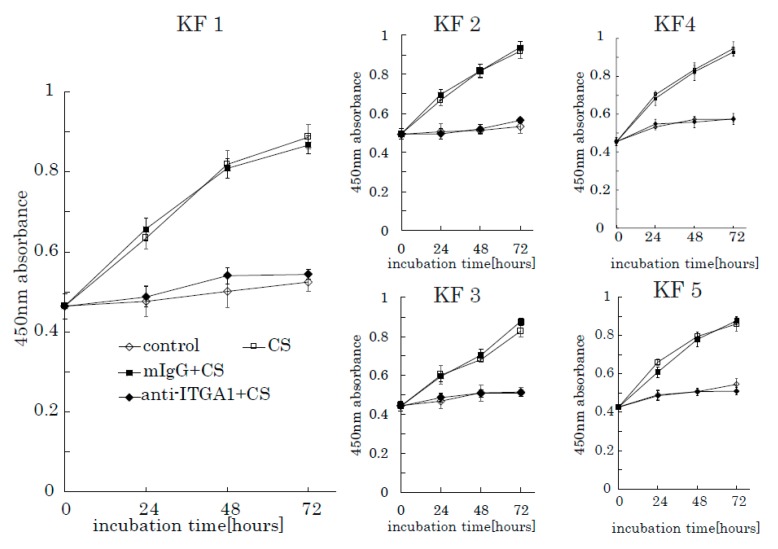
The anti-integrin α1 antibody blocks the CS-induced proliferation of KFs. Proliferation rates of KFs incubated with CS in the presence of an anti-integrin α1 antibody were analyzed. Mouse anti-human IgG was used as a control. Error bars represent the standard deviation (*n* = 3).

To further investigate the role of integrin α1 in CS-induced KF proliferation, we used an anti-integrin α1 antibody and mouse anti-human IgG as a control. When incubated with anti-integrin α1 antibodies, the stimulation effect of CS on KF proliferation was successfully blocked (Figure 8). Furthermore, anti-integrin α1 antibody inhibited AKT and FAK phosphorylation (Supplementary Figure S3). These results suggest that integrin α1 is a CS-receptor that initiates CS-stimulated KF proliferation through the activation of the FAK-AKT pathway.

## 3. Discussion

### 3.1. The Mechanism Underlying the Promotion of KF Proliferation by CS

In the present study, we revealed that the cell-proliferative effect of chondroitin sulfate (CS) in KFs in comparison to NFs. A tetrazolium salt assay and intracellular signaling pathway analysis revealed that the cellular proliferation of starved KFs was actively promoted. 

In keloids, growth factors, such as IGF-1 and TGFβ1, are closely associated with the pathogenesis of keloids, and fibroproliferation has been reported to be regulated—in part—by the ERK pathway, which is signaled by receptor tyrosine kinase phosphorylation by IGF-1. The PI3K-Akt signaling pathway was also found to be synchronously activated [9,20,22]. Thus, we initially hypothesized that CS stimulated keloid fibroblast proliferation through the ERK and Akt pathways by cooperating with the IGF1 receptor. However, the Akt pathway was activated by the addition of CS, while the ERK pathway was not. 

**Figure 9:**
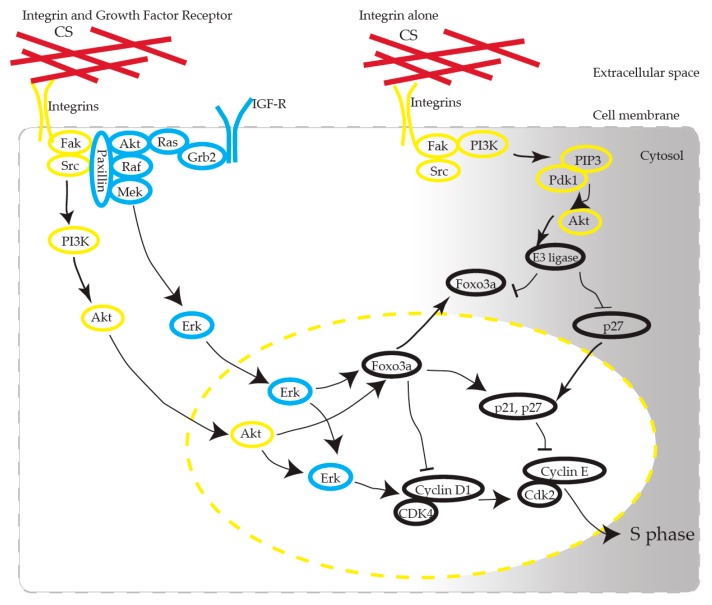
Possible pathways involved in CS-stimulated KF proliferation. CS interacts with integrin, induces AKT activation, and produces cell cycle progression. Note that Erk activation is mandatory for cell cycle progression. Arrows stand for activation; bars represent inhibition.

We propose that CS induces the activation of AKT in KFs via integrins (Figure 9). CS has been recognized as one of the scaffold materials and has no functional role as a ligand molecule in receptors. How CS, as a static ECM molecule, exhibits its functional effect has been argued [23]. CS interacts with various growth factors, including FGF-2, PDGF, and TGF-β, and BDNF, and the biological functions of CS are presumed to be partially revealed through these interactions [12]. CS-E, one of the CS subclasses characterized by the predominant disulfated disaccharide E unit (GlcUA-GalNac[4,6-O-disulfate]), possesses strong neuritogenic activity toward primary hippocampal neurons [24]. CS-E binds to several growth factors, such as midline (MK), brain-derived neurotrophic factor (BDNF), and stimulates neurite outgrowth through the activation of MK and BDNF signaling [25].

### 3.2. CS Is a Structural Molecule and a Regulator of Growth Factor Signaling and Neurite Outgrowth

Integrins are transmembrane receptors that activate intracellular signal transduction pathways, such as cell cycle regulation [26,27], organization of the intracellular skeleton, and receptor organization. FACS profiling has shown that α1 integrins are specifically elevated in keloids and hypertrophic scars [28], which is compatible with our results. Integrins communicate with growth factor receptors to control specific cellular responses to stimuli originating in the extracellular environment [29,30]. Integrins control growth factor receptor activity through the differential binding affinity of ECM ligands for integrins.

FAK is a 125 kDa non-receptor protein kinase that directly binds to the cytoplasmic tail of β-integrin and which plays a significant role in integrin-mediated signaling. The biological importance of FAK-mediated signal transduction is understood by the fact that it plays a fundamental role in embryonic development, control of cell migration, and cell cycle progression [31]. Recent studies have reported co-operation between integrins and growth factor receptors in ECM-originated cell cycle regulation—for example, cells detached from ECM or lacking integrins cannot proceed into the cell cycle, even in the presence of growth factors [32,33,34,35]. Integrins control the cell cycle via their activation of the adhesion-activated Akt pathway and the Erk pathway, which produces cyclin D1 activation via signal crosstalk, to allow cell cycle transition through the G1/S phase [36], which we observed in our experiment.

Marlene et al. reported [37] that KFs may have a lower inherent threshold for S phase entry and hypothesized that the threshold might occur due to elevated ERK activity. We consider that the CS-mediated proliferation that we observed in this study occurred through the following mechanism: KFs have a high tendency for proliferation (ERK phosphorylation), and CS stimulation triggers entry into the S phase via the AKT pathway.

Specific types of CS have been reported to be responsible for the pathogenesis of diseases in neural cells [38,39,40,41] and melanoma cell lines [42,43,44,45]. The CS used in our study included equal amounts of CS-A, CS-B, and CS-C, which is a similar composition to that in in vivo keloid tissue, as we previously reported. Further research is needed to reveal the type and amount of CS that are most responsible for KF proliferation.

These observations suggest that CS plays a fundamental role in KF proliferation as a ligand of integrins. We showed that integrin α1 is overexpressed in keloids, which was consistent with the previous report [28]. Integrin α1 might be a putative therapeutic target as the entrance of the CS-induced signaling pathway. Additionally, we observed that the ERK of KFs was highly activated in comparison to NFs. RT-PCR revealed that the IGR-1 receptor expression in keloids is upregulated in comparison to NFs [6,7,8,46]. The overexpression of IGF1-R might activate ERK in KFs (Figure 9) [36].

Rather than as a growth factor reservoir or a scaffold of cell attachment, CS itself had an inductive role in the cellular proliferation of KF. Although we demonstrated that CS played a role in the proliferation of KFs, we could not rule out the possibility that another pathway (besides the integrins) produces intracellular proliferative signals, like contactin-1 in neural cells [47]. The difference between the 2D in vitro culture model and the 3D real tissue-like microenvironment at the point of CS concentration and the difference in CS sulfation has not been sufficiently investigated, and further research may be needed.

Recently, Shida et al. [21] reported that CS-D induced neurite outgrowth of primary hippocampal neurons and that this process is mediated through the integrin αVβ3, which is tightly associated with activation of the FAK pathway. This is the first report to demonstrate that an integrin can function as a cell surface CS receptor. From our data, integrin α1 may play a critical role in the proliferation of KFs. However, we did not examine the direct binding of CS to integrin α1. Further investigations are required.

In conclusion, our data presented the mechanism responsible for the efficacy of CS degradation in keloid treatment [15]. The action of the integrin antibody against CS-stimulated cultures of KFs strongly suggests its potential application in the development of therapeutic approaches for keloids.

## 4. Materials and Methods

### 4.1. Tissue Specimens and Primary Cell Cultures and Treatments

Five patients with keloids (age, 33–84 years) and seven unrelated patients (age, 31–53 years) undergoing surgical treatment between September 2014 and July 2016 were enrolled in this study. The present study was performed with approval from the Institutional Reviewing Committee in Kyoto University Faculty of Medicine (G61, 14 December 2006), which adhered to the ethical standards as formulated in the Helsinki Declaration. Written informed consent was obtained from all patients. The skin tissue samples were obtained as surplus skin in plastic surgery. The sample information is shown in Table 1.

### 4.2. Fibroblast Cultures and the Cellular Proliferation Assay

Keloid fibroblasts and normal fibroblasts were extracted from surgical specimens by the explant method. Briefly, tissues were cut into 1 mm^3^ pieces, placed in plastic tissue culture dishes, and cultured in Dulbecco’s modified Eagle’s medium (DMEM; Sigma-Aldrich, St. Louis, MO, USA) supplemented with 10% fetal bovine serum (FBS), 10,000 U/mL penicillin G, and 10 mg/mL streptomycin sulfate. Cells were propagated at 37 °C, and semi-confluent cultures of fibroblasts were passaged by trypsinization up to two times before the analysis [1]. Fibroblasts were washed with warm phosphate-buffered saline (Takara Bio, Shiga, Japan) two times, trypsinized, and plated on PDL (poly-D-lysine)-coated 96-well cell plates (Corning Incorporated, New York, NY, USA) at a seeding density of 4000 cells per well. Fibroblasts were cultured in DMEM without FBS. After 24 h, the medium was changed, and starved cells were stimulated with CS (200 µg/mL). A colorimetric assay using a water-soluble tetrazolium salt WST-8 (Dojindo Laboratories, Kumamoto, Japan) was performed every 24 h to measure cell proliferation. Optical absorbance at a wavelength of 450 nm was measured using a VersaMax microplate reader (Molecular Devices, Sunnyvale, CA, USA). To inhibit the phosphorylation of PI3K (Phosphoinositide 3-kinase) by wortmannin (FUJIFILM Wako Pure Chemical, Osaka, Japan), pretreatment with or without wortmannin diluted in DMSO (10 nM), was performed 30 min before incubation with CS.

### 4.3. Western Blotting

Trypsinized and counted cells were seeded in 6-well poly-D-Lysine (PDL)-coated cells at a density of 400,000 cells per well. After incubation with CS, fibroblasts were washed twice with ice-cold phosphate-buffered saline (PBS), and then harvested with RIPA buffer (Nacalai Tesque, Kyoto, Japan) containing phosphatase and protease inhibitors. The cell lysate was sonicated and cleared by centrifugation (12,000 rpm, 4 °C, 20 min). The protein concentration of cell extracts was determined using a BCA protein assay kit (Thermo Fisher Scientific, Waltham, MA, USA). Cell lysate samples were separated by SDS-PAGE (gradient gels, 4%–12%) and then blotted onto 0.20 µm nitrocellulose membranes (Life Technologies, Carlsbad, CA, USA, Thermo Fisher Scientific) using the Xcell^®^ Blot module (Thermo Fisher Scientific). The membrane was blocked with 5% non-fat dry milk (Nacalai Tesque) in Tris-based saline containing 0.05% Tween 20 (TBST) for 1 h at room temperature and then incubated with primary antibodies (at the dilution described in Table 2) at 4 °C overnight. The membranes were washed with TBST and incubated with secondary antibodies conjugated with HRP (horseradish peroxidase). After washing as described above, the bound antibodies were visualized with an ECL kit according to the manufacturer’s instructions (Nacalai Tesque). Luminosity images were taken using LAS-3000 (Fujifilm, Tokyo, Japan). The antibodies that were used are listed in Table 2.

### 4.4. Real-Time PCR Analysis

Total RNA was extracted from cells using RNeasy Micro Kits (Qiagen, Hilden, Germany). First-strand cDNA was synthesized using a Prime Script RT reagent kit with a gDNA eraser (Takara Bio, Otsu, Japan). RT-PCR was performed with cDNA using a TaqMan Probe Assay (Thermo Fisher Scientific). Taqman Assay IDs Cyclin D1: Hs0076553_m1, integrin α 1: Hs00235006_m1, integrin α 2: Hs00158127_m1, integrin α 4: Hs00168433_m1, integrin α 5: Hs01547673_m1, integrin α v: Hs00233808_m1, integrin β 1: Hs01127536_m1, GAPDH: Hs02758991_g were used. The relative gene expression was normalized against an internal control, GAPDH. The expression of each gene was compared between depots using the ΔΔCt method.

### 4.5. The Analysis of the Effects of Anti-Integrin α1 Antibodies on KF Proliferation

Serum-free DMEM containing KFs prepared as described above was added to 96-well PDL-coated plates. After 24 h of incubation, an anti-integrin α1 antibody (MilliporeSigma, Burlington, MA, USA; MAB1973Z: 2 µg/mL) was added to the wells. Mouse anti-human IgG (Santa Cruz, SC-2025:0.1 mg/mL) was used as a control.

### 4.6. Other Reagents

Chondroitin sulfate (CS-A from whale cartilage, CS-B from pig skin, and CS-C from shark cartilage) was obtained from Seikagaku Biobusiness Corporation (Tokyo, Japan) under a Material Transfer Agreement between Kyoto University and Seikagaku Biobusiness Corporation. We applied CS as a mixture of equal amounts of CS-A, CS-B, and CS-C, which was a similar composition to that observed in keloids, as we previously reported [14]. All other reagents, if not stated, were obtained from Nacalai Tesque.

### 4.7. Data Analysis

Data were expressed as the mean ± SEM of at least three independent experiments. Unless otherwise stated, an ANOVA or two-tailed Student’s *t*-test were used to analyze the results. *P* values of < 0.05 were considered to indicate statistical significance.

## 5. Conclusions

The present study showed that CS strongly stimulates the signaling to induce KF proliferation via the AKT pathway, which was successfully inhibited by blocking PI3K. Anti-integrin α1 antibodies blocked the proliferative effect of CS on KF. These findings provide a reasonable explanation for the rapid proliferation of KFs in vivo, where accumulated CS is abundant. We conclude that the excessive accumulation of CS and the elevated expression of α1 bata1 integrin and IGF-1R have a synergistic effect on KF proliferation and the overproduction of ECM components, such as CS, which plays an essential role in the etiology of keloids. The CS-enriched extracellular milieu may interact with KF and vice versa resulting in the deposition of CS into ECM. We consider CS to be a key molecule in the etiology of keloids. Further study is required because our experiment only used a 2-D in vitro model. The positive effect on fibroblast proliferation may increase in 3-D cultures.

In conclusion, the KF-specific role of CS revealed in the present study represents a significant advance in the understanding of ECM-triggered proliferation, and has the potential to be applied in the development of treatments for keloids.

## Figures and Tables

**Table 1:** Characteristics of the patients enrolled in this study.

Keloid	Age (Years)	Sex	Location
K1	37	Female	abdomen
K2	34	Female	chest
K3	62	Male	abdomen
K4	69	Male	chest
K5	27	Female	shoulder
**Normal Skin**	**Age (Years)**	**Sex**	**Location**
N1	52	Female	chest
N2	53	Female	chest
N3	40	Female	abdomen
N4	31	Female	abdomen
N5	52	Female	chest
N6	28	male	abdomen
N7	11	male	abdomen

**Table 2:** Antibodies Used in the Study.

Antibody	Raised Species	Isotype	Clone	Dilution	Product Code	Source	Used In
phopho-AKT	rabbit monoclonal	IgG	D9E	1:2000	4060	Cell Signaling Technology	WB
total-AKT	rabbit monoclonal	IgG	C67E7	1:2000	4691	Cell Signaling Technology	WB
phospho-ERK	rabbit monoclonal	IgG	D13.14.4E	1:2000	4370	Cell Signaling Technology	WB
total-ERK	rabbit monoclonal	IgG	137F5	1:2000	4695	Cell Signaling Technology	WB
phospho-CDK2	rabbit polyclonal	-	-	1:500	2561	Cell Signaling Technology	WB
total-CDK2	rabbit monoclonal	IgG	78B2	1:500	2947	Cell Signaling Technology	WB
p21	rabbit monoclonal	IgG	12D1	1:500	2947	Cell Signaling Technology	WB
phospho-FAK	rabbit monoclonal	IgG	D20B1	1:500	8556	Cell Signaling Technology	WB
total-FAK	rabbit monoclonal	IgG	D2R2E	1:1000	13,009	Cell Signaling Technology	WB
GAPDH	mouse monoclonal	IgG	6C5	1:2000	sc-32233	Santa Cruz Biotechinology	WB
anti-mouse	goat	HRP-Linked IgG		1:2000	sc-2005	Santa Cruz Biotechinology	WB
anti-goat	donkey	HRP-Linked IgG		1:2000	sc-2020	Santa Cruz Biotechinology	WB
anti-rabbit	goat	HRP-Linked IgG		1:2000	sc-2004	Santa Cruz Biotechinology	WB
anti-rabbit	donkey	HRP-Linked IgG		1:2000	NA934	GE Healthcare Japan	WB
integrin α1	Mouse monoclonal	IgG1	FB12	1:500	MAB1973Z	Merch Millipore	C
-	mouse	IgG	-	1:250	SC-2025	Santa Cruz Biotechnology	C
			WB; Western Blotting, C; Cell Proliferation Assay				

## Supplementary Materials

Supplementary materials can be found at https://www.mdpi.com/1422-0067/21/6/1955/s1.
