# Supplementary material for: Chondroitin Sulfate Promotes the Proliferation of Keloid Fibroblasts Through Activation of the Integrin and Protein Kinase B Pathways
Source: Int J Mol Sci. 2020 Mar 13;21(6):1955. doi: 10.3390/ijms21061955 (PMC7139995; doi:10.3390/ijms21061955)
Supplement: Supplementary file 1 [file ijms-21-01955-s001.pdf]

International Journal of
Molecular Sciences

MDPI

Supplementary Figures

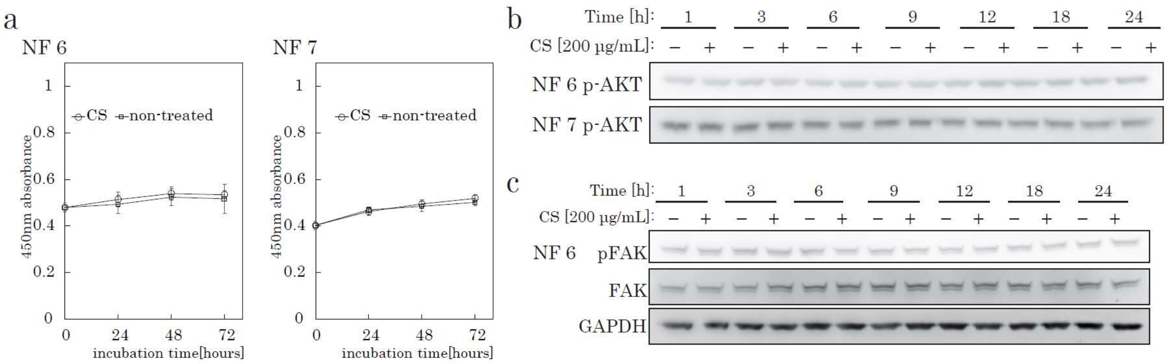

Supplementary Figure S1. (a). CS does not stimulate normal fibroblast (NF) proliferation. NFs were cultured with chondroitin sulfate (CS) or without CS. Cell proliferation was analyzed using a colorimetric assay with a water-soluble tetrazolium salt as the substrate. Proliferation curves of NFs (N6 and N7, Table 1) are shown. Error bars represent the standard deviation (n=3). (b). Western blotting of proteins regulating the intracellular signaling pathway in NFs (N6 and N7). NFs were treated with CS for up to 24 hours. NFs display no response in the phosphorylation of AKT and FAK after CS stimulation.

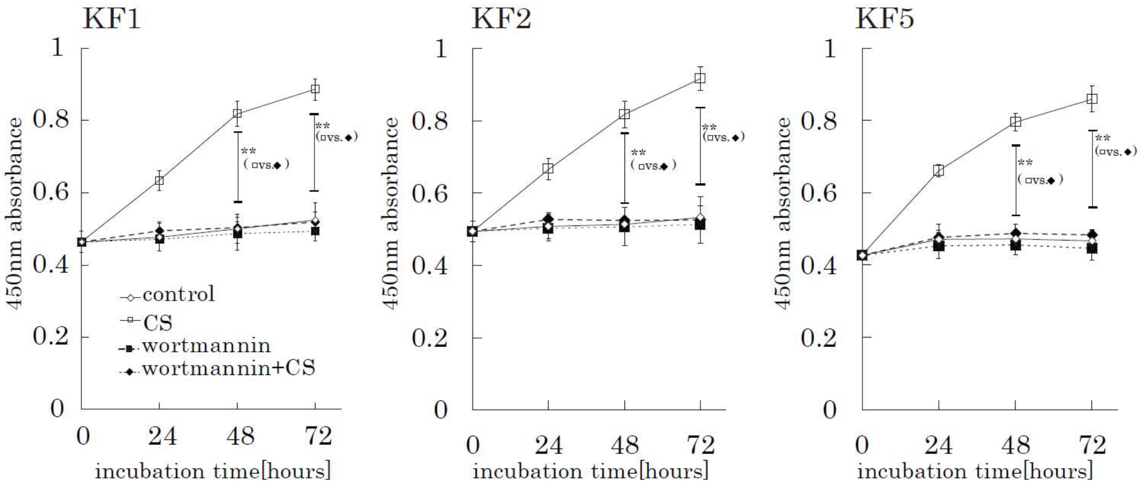

Supplementary Figure S2. Wortmannin inhibits CS-induced activation of KF proliferation. KFs were cultured with wortmannin or without wortmannin, in the presence or absence of CS. Cell proliferation was analyzed using a colorimetric assay with a water-soluble tetrazolium salt as the substrate. Proliferation curves of KFs are shown. Error bars represent the standard deviation (n=3). ** $p<0.01$.

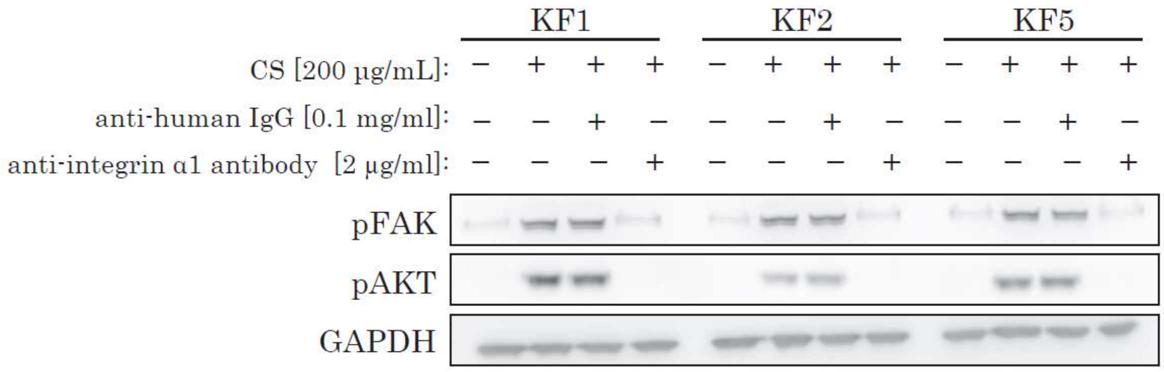

Supplementary Figure S3. The anti-integrin α1 antibody blocks the CS-induced activation of AKT and FAK. KFs were incubated with CS and anti-integrin α1 antibody and analyzed by Western blotting. Mouse anti-human IgG was used as a control.
